# Early detection of multidrug- and pre-extensively drug-resistant tuberculosis from smear-positive sputum by direct sequencing

**DOI:** 10.1186/s12879-017-2409-6

**Published:** 2017-04-24

**Authors:** Jun Chen, Peng Peng, Yixiang Du, Yi Ren, Lifeng Chen, Youyi Rao, Weihua Wang

**Affiliations:** 1 Department of Laboratory Medicine, Wuhan Institute for Tuberculosis Control, Wuhan Pulmonary Hospital, Wuhan, 430030 China; 2 Department of Internal Medicine, Wuhan Institute for Tuberculosis Control, Wuhan Pulmonary Hospital, Wuhan, 430030 China; 3 Department of Tuberculosis Control, Wuhan Institute for Tuberculosis Control, Wuhan Pulmonary Hospital, Wuhan, 430030 China

**Keywords:** *Mycobacterium tuberculosis*, MDR-TB, Pre-XDR-TB, Rifampicin, Isoniazid, Fluoroquinolone

## Abstract

**Background:**

Emergence of multidrug- and extensively drug-resistant tuberculosis (M/XDR-TB) is a major hurdle for TB control programs especially in developing countries like China. Resistance to fluoroquinolones is high among MDR-TB patients. Early diagnosis of MDR/pre-XDR-TB is essential for lowering transmission of drug-resistant TB and adjusting the treatment regimen.

**Methods:**

Smear-positive sputum specimens (*n* = 186) were collected from Wuhan Institute for Tuberculosis Control. The DNA was extracted from the specimens and run through a Sanger sequencing assay to detect mutations associated with MDR/pre-XDR-TB including the *rpoB* core region for rifampicin (RIF) resistance; *katG* and *inhA* promoter for isoniazid (INH) resistance; and *gyrA* for fluoroquinolone (FQ) resistance. Sequencing data were compared to phenotypic Lowenstein-Jensen (L-J) proportion method drug susceptibility testing (DST) results for performance analysis.

**Results:**

By comparing the mutation data with phenotypic results, the detection rates of MDR-TB and pre-XDR-TB were 84.31% (43/51) and 83.33% (20/24), respectively. The sequencing assay illustrated good sensitivity for the detection of resistance to RIF (96.92%), INH (86.89%), FQ (77.50%). The specificities of the assay were 98.35% for RIF, 99.20% for INH, and 97.26% for FQ.

**Conclusions:**

The sequencing assay is an efficient, accurate method for detection of MDR-TB and pre-XDR-TB from clinical smear-positive sputum specimens, should be considered as a supplemental method for obtaining early DST results before the availability of phenotypic DST results. This could be of benefit to early diagnosis, adjusting the treatment regimen and controlling transmission of drug-resistant TB.

## Background

The emergence and spread of multidrug-resistant tuberculosis (MDR-TB) and extensively drug-resistant tuberculosis (XDR-TB) has become a major public health threat worldwide. According to the recent report of World Health Organization (WHO), globally, an estimated 3.3% of new cases and 20% of previously treated cases have MDR-TB; these levels have remained virtually unchanged in recent years [1]. In 2014, there were an estimated 480,000 new cases of MDR-TB worldwide, and approximately 190,000 deaths from MDR-TB. Among patients with pulmonary TB who were notified in 2014, an estimated 300,000 had MDR-TB. More than half of these patients were in India, China and the Russian Federation. On average, an estimated 9.7% of people with MDR-TB have XDR-TB [1]. In 2007–08, a nationwide drug-resistance survey conducted in China showed that the proportion of MDR-TB in smear-positive pulmonary TB patients was 8.32%. Of these, the proportion of MDR-TB in new patients and re-treated patients was 5.71 and 25.64%, respectively [2].

Pre-XDR-TB denotes resistance to rifampicin and isoniazid, as well as to at least one fluoroquinolone (FQ) or 1 second-line injectable drug: kanamycin, amikacin or capreomycin. FQs have been proven to be among the most effective second-line anti-mycobacterial drugs and are recommended for the treatment of MDR-TB [3]. When patients are infected with FQ-resistant MDR-TB strains, the treatment regimen needs to be adjusted and the prognosis is poor [4]. In China, among patients with MDR tuberculosis, 24.9% of those with new cases of tuberculosis and 27.5% of those with previously treated tuberculosis had resistance to ofloxacin [2]. In India, 29% of patients with previously treated tuberculosis had resistance to ofloxacin [5]. The rates were as high as 60.6 and 78.3% in two hospital-based studies in Shandong province and Shanghai [6, 7]. Therefore, effective and accurate diagnosis of these MDR-TB and pre-XDR-TB patients is urgently needed for choosing a reasonable regimen and preventing transmission.

Conventional TB diagnosis depends on culture growth of mycobacteria, which is very slow, time-consuming. Rapid molecular diagnostics, based on the detection of specific mutations that confer drug resistance, provide approaches to identify drug resistance in *Mycobacterium tuberculosis* (MTB). These technologies include the Hain MTBDRplus line probe assay, the genechip assay and the GeneXpert MTB/RIF assay [8–10]. However, these methods have some limitations, they are unable to distinguish silent mutations from mutations associated with drug resistance, and there has been little progress toward the broad application of these molecular diagnostics for the simultaneous detection mutations associated with resistance to the first-line and second-line anti-tubercular drugs [11–13]. In order to discover MDR-TB and pre-XDR-TB patients, we report an effective method which is able to detect MDR-TB and pre-XDR-TB from smear-positive sputum by direct sequencing.

## Methods

### Source of sputum specimens and the standard strain

From February 2014 to January 2015, among 1233 suspected TB patients from Department of internal medicine in Wuhan Institute for Tuberculosis Control,Wuhan Pulmonary Hospital, 207 smear-positive sputum specimens were acquired, of which, 7 specimens were culture-negative, 5 specimens were contaminated, non-tubercular mycobacteria (NTM) were isolated from 4 specimens, 5 specimens were also excluded due to no amplification of four resistance-associated mutation genes, finally 186 sputum specimens were enrolled in this study. The 186 specimens came from 186 different patients: 91 initial treatment patients and 95 retreatment patients. The smear-positive sputum specimens were identified as MTB by detecting IS6110 positive by real-time PCR (Daan gene, Guangzhou, China). *Mycobacterium tuberculosis* standard strain (H37Rv, ATCC27294) was purchased from the National Culture Collection (Beijing, China).

### Specimen processing and culture of *Mycobacteria*

Every sputum specimen was decontaminated with a 2–4 fold volume of 4% NaOH, liquefied at room temperature for 20 min. The processed specimen was inoculated on to Lowenstein-Jensen (L-J) based solid medium for isolation of Mycobacteria. The duration of incubation for L-J solid culture was 56 days.

### Genomic DNA extraction

Transfer 1.0 ml above liquefied sputum to a 1.5 ml tube and centrifuge in a tabletop centrifuge at 6000 g for 15 min at room temperature. The supernatant was discarded carefully and the sediment was washed with 0.9% NaCl solution and recentrifuged as before. The pellet was washed again. Next, 50ul lysis solution was added to the processed sediment, mixed well and incubated at 100 °C for 10 min. The lysate was stored at 4–8 °C until use.

### Design of primers and PCR amplification

Amplification and sequencing primers of *rpoB* gene were described in a previous study [14]. The primers of *katG*, *inhA* promoter and *gyrA* genes were designed with Primer Premier software. Primer Pair Specificity was detected by Primer-BLAST [15]. Primers and amplicon sizes were presented in Table 1. Amplification reaction were performed in a total volume of 30 μl consisting of 15 μl 2× PCR MIX (Fermentas), 3 μl 2 μmol/L each primer, 2 μl DNA lysate and 10 μl ddH_2_O. Thermo-cycling conditions were as follows: 95 °C for 2 min followed by 40 cycles of 35 s at 95 °C, 30 s at 55 °C, 45 s at 72 °C and final elongation at 72 °C for 5 min.

**Table 1:** Primers for polymerase chain reaction amplification and sequencing

Gene	Primer	Sequence	Size (bp)
*rpoB*	Forward	CCACCCAGGACGTGGAGGCGATCACACCG	329
	Reverse	CGTTTCGATGAACCCGAACGGGTTGAC	
*katG*	Forward	GGGGCTGATCTACGTGAACC	363
	Reverse	CTCTTCGTCAGCTCCCACTC	
*inhA* promoter	Forward	ATGGAAGGCAGAAGCCGAGTA	398
	Reverse	GACTGAACGGGATACGAATGG	
*gyrA*	Forward	CAGCGCAGCTACATCGACTA	356
	Reverse	CTCAGCATCTCCATCGCCAA	

### Sequencing of *rpoB*、*katG*、*inhA* promoter and *gyrA* genes

PCR products were purified with UNIQ-10 column purification kit (Sangon, Shanghai, China) and then sequenced with an automatic DNA sequencer (Applied Biosystems 3730xl DNA analyzer, USA). Mutations in *rpoB*、*katG*、*inhA* promoter and *gyrA* genes were identified using BLASTn (https://blast.ncbi.nlm.nih.gov/blast.cgi) (version number: BLAST2.2.31) by comparison with *Mycobacterium tuberculosis* wild-type strain H37Rv sequence. The mutations were compared with the TB Drug Resistance Mutation Database (https://tbdreamdb.ki.se/Info/) [16]. Sequences matching mutations previously associated with resistance were considered genotypically resistant, whereas wild-type sequences were considered genotypically susceptible, and those not matching resistance-associated mutations or wild-type sequences were considered genotypically indeterminate for the given gene regions [17].

### DST-drug susceptibility tests of *Mycobacterium tuberculosis* by L-J proportion method

Cultures obtained on L-J medium were collected and tested susceptibility for rifampicin, isoniazid and ofloxacin. DST was performed by L-J proportion method. The critical drug concentrations were 40 μg/ml for rifampicin, 0.2 μg/ml for isoniazid and 2 μg/ml for ofloxacin. The standard strain H37Rv was used as control. External quality assurance on DST by proficiency testing was conducted regularly (once a year) by the national reference laboratory of China.

### Statistical analysis

SPSS 22.0 software (SPSS Inc., Chicago, IL, USA) was used for the statistical analysis. The sensitivity and specificity were calculated for sequencing results versus L-J proportion method, which was considered as the gold standard of DST.

## Results

### Detection of MDR-TB and pre-XDR-TB by directing sequencing

Among the 186 clinical isolates, no mixed culture was isolated, 105 isolates were susceptible to all of three drugs: RIF, INH and OFX. 51 isolates were MDR-TB strains, of which 24 isolates were pre-XDR-TB strains. The detection rates of MDR-TB and pre-XDR-TB by direct sequencing were 84.31% (43/51) and 83.33% (20/24), respectively.

### Sequencing results for the detection of gene mutations

The mutations of the 186 smear-positive sputum specimens in *rpoB*, *katG*, *inhA* promoter and *gyrA* genes were shown in Table 2. Of the 186 smear-positive sputum specimens, the sequencing identified 13 types of mutations in rifampicin resistance-determining region (RRDR) of the *rpoB* gene. The mutation rates of codon 531 and 526 were 67.69% (44/65) and 16.92% (11/65), respectively. One specimen had double mutation, and 2 specimens had base deletion. There were 2 types of mutations in *katG* gene, which were in codon 315. There were 4 types of mutations in *inhA* promoter, C(−15) → T mutation (10/14) was predominant. Among 54 specimens related to INH resistance, 75.93% (41/54) of the specimens had only *katG* gene mutation, 16.67% (9/54) had only *inhA* promoter mutation, 7.41% (4/54) had *katG* gene and *inhA* promoter mutations. There were 7 types of mutations in *gyrA* gene. The mutation rates of codon 94 and 90 were 68.57% (24/35) and 20.00% (7/35), respectively. Three heteroresistant specimens were detected by Sanger sequencing plots, 2 with the *katG* S315 T mutation, 1 with the *rpoB* S531 L mutation. All 3 specimens came from retreatment patients.

**Table 2:** Mutation distribution of *rpoB*, *katG*, *inhA* promoter and *gyrA* genes in the smear-positive sputum specimens

Drugs	Genes	Nucleotide change	Amino acid change	Number(%)	Number of phenotypically resistant strains
rifampicin	*rpoB* ^a^	G(1546) → T	Asp(516) → Tyr	2(1.59)	1
		A(1547) → T	Asp(516) → Val	1(1.59)	1
		A(1547) → T;A(1552) → G^b^	Asp(516) → Val; Asn(518) → Asp	1(1.59)	1
		AGA(1550–1552)deletion^b^	Frame shift	1(1.59)	1
		C(1576) → T	His(526) → Tyr	2(2.18)	2
		C(1576) → A	His(526) → Asn	2(2.18)	1
		C(1576) → G	His(526) → Asp	1(1.59)	1
		A(1577) → T	His(526) → Leu	4(6.35)	4
		A(1577) → G	His(526) → Arg	1(1.59)	1
		A(1577) → C	His(526) → Pro	1(1.59)	1
		C(1592) → T	Ser(531) → Leu	44(68.25)	44
		T(1598) → C	Leu(533) → Pro	4(6.35)	4
		C(1542) → T;ATGGAC(1543–1548)deletion^b^	Frame shift	1(1.59)	1
isoniazid	*katG*	G(944) → C	Ser(315) → Thr	40(95.24)	40
		G(944) → A	Ser(315) → Asn	2(4.76)	2
	*inhA* promoter	T(−8) → C		1(7.69)	1
		C(−15) → T		10(76.92)	9
		G(−17) → T		1(7.69)	1
		C(−34) → T^c^		1(7.69)	1
ofloxacin	*gyrA*	C(269) → T	Ala(90) → Val	5(14.29)	4
		C(269) → G	Ala(90) → Gly	2(5.72)	1
		T(271) → C	Ser(91) → Pro	4(11.43)	4
		G(280) → T	Asp(94) → Tyr	4(11.43)	3
		G(280) → A	Asp(94) → Asn	5(14.29)	4
		A(281) → G	Asp(94) → Gly	12(34.29)	12
		A(281) → C	Asp(94) → Ala	3(8.57)	3

^a^For rpoB gene, E coli codon numbering system is used

^b, c^these mutations were not found in the TB Drug Resistance Mutation Database (https://tbdreamdb.ki.se/Info/)

### The performance of direct sequencing for detection of MTB resistance

Compared with L-J proportion method, direct sequencing showed higher sensitivities in RIF and INH than that in OFX. Specificities were very high in three drugs. The performance of direct sequencing for detection of MTB resistance was summarized in Table 3.

**Table 3:** Performance of direct sequencing in detecting of resistance of three antitubercular drugs

Drugs	Gene targets	No.(%) of strains/total no. of strains			% sensitivity	% specificity
		Phenotypically resistant	Phenotypically resistant with mutations	Phenotypically susceptible with mutations		
rifampicin	*rpoB*	65/186(34.95)	63/65(96.92)	2/121(1.65)	96.92	98.35
isoniazid	*katG*, *inhA* promoter	61/186(32.80)	53/61(86.89)	1/125(0.80)	86.89	99.20
ofloxacin	*gyrA*	40/186(21.51)	31/40(77.50)	4/146(2.74)	77.50	97.26

## Discussion

Early diagnosis of MDR/pre-XDR-TB is crucially important for both working out a rational regimen and preventing the transmission of them. In this study, the drug-resistant loci of *rpoB*, *katG*, *inhA* promoter and *gyrA* genes were amplified and sequenced to detect mutations from smear-positive sputum. The entire procedure, from extracting DNA to reporting results, can be accomplished within 3 days, but the traditional culture method and DST needs 1 to 3 months. This shortened the turnaround time of DST. Direct sequencing in our study was able to detect not only MDR-TB, but also pre-XDR-TB, However, GeneXpert MTB/RIF assay and Hain MTBDRplus can only detect MDR-TB, not pre-XDR-TB. Zakham F et al. [18] also detected only MDR-TB by direct sequencing. Although pyrosequencing can be used to detect XDR-TB, fragments for pyrosequencing are short and more primer pairs are needed for amplifying long fragments, such as *rpoB* gene [17], at the same time, pyrosequencing applies to the sputum specimens of 1+ or 2+ acid-fast staining, the nested PCR might be essential [19]. Whole genome sequencing provides more information of resistance and helps explore new mechnisms of drug resistance, but this method is expensive and mainly used to sequence MTB strains [20].

Our results showed that the detection rates of MDR-TB and pre-XDR-TB were 84.31% (43/51) and 83.33% (20/24), respectively. It was similar to MTBDRplus assay [8, 14]. The detection rate (84.31%) of MDR-TB was slightly lower that reported in previous study from Shanghai (88%) [21]. It was possible that the mutations in *katG* and *inhA* promoter of 8 MDR-TB specimens were not detected by direct sequencing in our study. This indicated the complexity of INH resistance, and there may exist mutations in other loci such as *ahpC* gene [22]. 47.06% (24/51) MDR-TB patients were pre-XDR-TB, which was much higher than that of the study by Zhao Y et al. [2]. The reason might be related to the source of our patients. Our hospital is a specialized hospital for pulmonary diseases. The patients mainly came from the different areas of Hubei province, suffered from severe TB and were treated repeatedly. The result of this study is in agreement with that of previous hospital-based studies [6, 7].

Mutations in a defined region of the 81 base pair (bp) region of the *rpoB* gene account for more than 95% of RIF resistance. Our results showed mutations at positions 531, 526 and 516 were among the most frequent mutations in RIF-resistant strains, which is in agreement with previous studies [23, 24]. Two *Mycobacterium tuberculosis* strains had deletion mutations. Four had mutation in codon 533, which were resistant to RIF. Although whether mutation in codon 533 is related to RIF resistance or not is disputed [8, 25, 26], our results indicated that mutation in codon 533 might cause RIF resistance. In RIF assay, the sensitivity and specificity of the direct sequencing were 96.92 and 98.35%, respectively. We could not detect mutation in 2 phenotypically RIF-resistant strains by this study, which could be due to the occurrence of mutations outside the RRDR or other loci in the genome. Direct sequencing detected mutations in 2 specimens that were determined to be phenotypically susceptible. The mutation of one specimen was detected at codon Asp 516 Tyr, the other at codon His 526 Asn. Reportedly, specific mutations in codons 511, 516, 518 and 522 are associated with lower-level resistance to RIF [27].

The *katG* S315 T mutation is the most common mutation in INH-resistant strains. Resistance to INH can also occur by mutations in the promoter region of *mabA*/*inhA* operon, causing overexpression of *inhA*, or by mutations at the *inhA* active site, lowering the *inhA* affinity to the INH-NAD adduct. Mutations in *inhA* or its promoter region are usually associated with low-level resistance and are less frequent than *katG* mutations [28]. Our results showed that *katG* gene mutation is the main mutation in INH-resistant strains, followed by *inhA* promoter. Combination of *katG* gene with *inhA* promoter increased the INH resistance detection by 16.67% than only detection of *katG* gene. This is in agreement with the report by Seifert M [29]. We found that the *katG* S315 T mutation from two retreat patients was heteroresistant by Sanger sequencing plot, this indicated that heteroresistance might develop during repeated treatment. In INH assay, the sensitivity and specificity of the direct sequencing were 86.89 and 99.20%, respectively. There may be mutations in other loci such as *ahpC* gene [22].

In the present study, *gyrA* mutations were predominantly found to occur in codons 90, 91, and 94. D94G and A90V were the most common mutations, largely corroborating the findings of our previous study [30] and some other studies [31–33]. In OFX assay, the sensitivity and specificity of the direct sequencing were 77.50 and 97.26%, respectively. In the OFX-resistant MTB clinical isolates, 31 of 40 (77.50%) had mutations in quinolone resistance-determining region (QRDR) of *gyrA* gene and the other nine strains had no mutations in *gyrA*. Sequencing *gyrB* gene, especially in MDR-TB patients, increased the sensitivity for FQ resistance by 12% [34]. Other resistance mechanisms, such as decreased cell-wall permeability to the drug and efflux pumps probably accounted for the FQ resistance in other FQ-resistant isolates without *gyrA* and gyrB mutations [35]. Further studies are needed to improve the sensitivity for FQ resistance. The total mutation frequency of *gyrA* gene in this study was similar to that in Guangdong province (73.3%) [36] and that in France (78%) [34]. Although the total sensitivity of OFX resistance was low (77.50%), we found that the detection rate of pre-XDR-TB by direct sequencing were 83.33% (20/24), this would help adjust the treatment regimen for those pre-XDR-TB patients.

There are some limitations in our study. Direct sequencing was mainly applied to smear-positive specimens. There were not enough amplification templates for direct sequencing when smear-negative specimens were used. We amplified different concentrations of H37Rv strain, more than 10^4^ copies/ml could be amplified and used for direct sequencing. Smear-positive NTM specimens were not amplified, amplification of IS6110 might be needed to distinguish MTB from NTM.

## Conclusions

In conclusion, direct sequencing is an efficient and accurate method for detecting MDR/pre-XDR-TB from smear-positive sputum specimens. Although there is room to improve the sensitivity of the assay, our results indicate that direct sequencing should be considered a supplemental method for obtaining early DST results before the availability of phenotypic DST results. This could be of benefit to early diagnosis, adjusting the treatment regimen and controlling transmission of drug-resistant TB.

## Abbreviations

*ahpC*: Alkyl hydroperoxide reductase gene
FQ: Fluoroquinolone
*gyrA*: Gyrase A subunit gene
INH: Isoniazid
*inhA*: NADH-dependent enoyl-ACP reductase gene
*katG*: Mycobacterial catalase-peroxidase gene
MDR-TB: Multidrug resistant tuberculosis
OFX: Ofloxacin
pre-XDR-TB: Pre-extensively drug-resistant tuberculosis
RIF: Rifampicin
*rpoB*: the β subunit of RNA polymerase gene
TB: Tuberculosis
XDR-TB: Extensively drug-resistant tuberculosis
